# DRAMP 3.0: an enhanced comprehensive data repository of antimicrobial peptides

**DOI:** 10.1093/nar/gkab651

**Published:** 2021-08-14

**Authors:** Guobang Shi, Xinyue Kang, Fanyi Dong, Yanchao Liu, Ning Zhu, Yuxuan Hu, Hanmei Xu, Xingzhen Lao, Heng Zheng

**Affiliations:** School of Life Science and Technology, China Pharmaceutical University, Nanjing 211100, P.R. China; School of Life Science and Technology, China Pharmaceutical University, Nanjing 211100, P.R. China; School of Life Science and Technology, China Pharmaceutical University, Nanjing 211100, P.R. China; School of Life Science and Technology, China Pharmaceutical University, Nanjing 211100, P.R. China; School of Life Science and Technology, China Pharmaceutical University, Nanjing 211100, P.R. China; School of International Pharmaceutical Business, China Pharmaceutical University, Nanjing 211100, P.R. China; The Engineering Research Centre of Peptide Drug Discovery and Development, China Pharmaceutical University, Nanjing 211100, P.R. China; School of Life Science and Technology, China Pharmaceutical University, Nanjing 211100, P.R. China; School of Life Science and Technology, China Pharmaceutical University, Nanjing 211100, P.R. China

## Abstract

Stapled antimicrobial peptides are an emerging class of artificial cyclic peptide molecules which have antimicrobial activity and potent structure stability. We previously published the Data Repository of Antimicrobial Peptides (DRAMP) as a manually annotated and open-access database of antimicrobial peptides (AMPs). In the update of version 3.0, special emphasis was placed on the new development of stapled AMPs, and a subclass of specific AMPs was added to store information on these special chemically modified AMPs. To help design low toxicity AMPs, we also added the cytotoxicity property of AMPs, as well as the expansion of newly discovered AMP data. At present, DRAMP has been expanded and contains 22259 entries (2360 newly added), consisting of 5891 general entries, 16110 patent entries, 77 clinical entries and 181 stapled AMPs. A total of 263 entries have predicted structures, and more than 300 general entries have links to experimentally determined structures in the Protein Data Bank. The update also covers new annotations, statistics, categories, functions and download links. DRAMP is available online at http://dramp.cpu-bioinfor.org/.

## INTRODUCTION

Antimicrobial resistance (AMR), declared by the WHO as one of the top 10 global public health threats facing humanity, threatens the effective prevention and treatment of an ever-increasing range of infections caused by bacteria, parasites, viruses and fungi (1). It is projected that antibiotic-resistant bacteria may kill 30 million people by 2050 (2). In hospital settings, research and development of new antimicrobial medicines, vaccines and diagnostic tools are urgently needed. Antimicrobial peptides (AMPs) are structurally variable short peptides with a broad spectrum of antimicrobial activities that have a key role in general defence mechanisms against microbial pathogens in all classes of life and can be viewed as stemming the rising tide of antibiotic resistance (3,4). Since the first AMP extracted from the skin secretions of speckled frogs was described by Kiss and Michl in the 1960s, researchers have achieved substantial progress in the isolation and synthesis of new AMPs, as well as understanding structure-activity relationships (5–7). Detailed data, such as sequence, structure, physicochemical properties, and biological activity, can accelerate the discovery of AMPs. With the increasing need for systematic annotation, various AMP databases have been created and mentioned in the literature. APD (8) is a well-known AMP database focused on natural AMPs, which includes a total of 2619 manually curated AMPs, as well as rich annotations and classifications. CAMP (9) is characterized by its large numbers of sequences and family-specific sequence signatures. DBAASP (10), a database presenting its data in a table-structured way, from which users can accurately retrieve the information they need and easily access molecular dynamics structural models. dbAMP (11) and LAMP (12) have also been developed for their generality, whereas other public AMP databases, such as CancerPPD (13), Hemolytik (14), THPdb (15), InverPep (16) and AntiTbPdb (17), are specialized with respect to their information characteristics.

Natural AMPs are traditional and seemingly sound choices for clinical trials and practical applications. However, the structural characteristics of such peptides are often not stable in vivo, and further application of peptides as medicine is seriously hindered by their poor pharmacokinetic features (18). Therefore, peptides with novel topological structures may overcome the limitation of instability. ‘Stapling’ is a strategy of macrocyclization that covalently tethers the sidechain of natural or non-natural amino acids to permanently reinforce a particular cyclic restricted structure (19,20). Hydrocarbon stapling has been applied to stable the helices targeting protein interactions, with a lead anticancer stapled peptide goes into phase II trials (21). However, early attempts to install staples into AMP sequences generated constructs with optimized membrane permeability and metabolic stability but variable antimicrobial activity and unselective membrane lysis, with high hemolytic effect and low therapeutic index (22–25). In 2019, Mourtada *et al.* reported an algorithm for design stale, protease-resistant, potent and nontoxic stapled AMPs, based on structure-function-toxicity relationship studies on stapled magainin II (26). Over the past several years, there are also a plethora of other articles describing low-toxic stapled peptides which have been published. A comprehensive dataset including such peptides will contribute to design of stapled AMPs with better draggability. To our knowledge, to date, no heed has been paid by any database to collecting and organizing the scattered stapled AMPs with information on experimentally determined antimicrobial and haemolytic activity. In the present study, for the first time, a systematic attempt has been made to collect this information, and it has been included in DRAMP.

DRAMP was first launched in 2016 and updated once in 2019 as a manually annotated database of AMPs, devoting attention to collecting natural, synthetic, patent, and clinical AMPs and providing general, structural, activity and literature information (27,28). Compared to the previous version, DRAMP 3.0 has more than 2300 additional entries to the previous 19 899 entries. Specifically, 181 stapled peptides have been added as unique features, and the other 2000+ entries are categorized as natural, synthetic and patent AMPs. New annotations, such as chemical modifications, interchain bonds, stereochemistry and cytotoxicity, have also been added. It is remarkable that DRAMP provides users rich charts and statistics for users to make full use of the database, and users can also submit new entries to expand the database. In addition, all download links directed to the latest AMP data resource have been renewed so that researchers can analyse our data expediently in their own way. Here, we further cover the updates and features of DRAMP 3.0, which may serve as a helpful resource for AMP study and design.

## MATERIALS AND METHODS

### Data collection

Experimentally validated AMPs in DRAMP are identified from peer-reviewed papers and patents. AMPs with the following characteristics are considered to meet DRAMP requirements: (i) each sequence is less than 100 amino acids in length separately; (ii) they have unambiguous mature sequences in which precursor and signal regions have been removed. We retrieved papers and patents including potential AMPs from PubMed (29), Web of Science, Lens, ClinicalTrials.gov (30) and chinadrugtrials.org.cn that met these criteria using keywords such as ‘antimicrobial peptides’, ‘host defence peptides’, ‘antibacterial peptides’, ‘antifungal peptides’, ‘antiviral peptides’, ‘anticancer peptides’, ‘antitumor peptides’, ‘stapled antimicrobial peptides’ and ‘screened antimicrobial peptides’. Literature including such terms was manually read and analysed to extract original AMP data. UniProt (31) and the Protein Data Bank (PDB) (32) were also queried to identify functional information and experimentally determined 3D structures. The sequences were divided into a general dataset, a patent dataset and a clinical dataset based on their source of references. Each entry of different datasets contains different annotations (28). Specific data were separated from the general dataset and currently consist of stapled peptides and candidate AMPs.

### Database construction and maintenance

Construction of DRAMP 3.0 was based on the Linux operating system (version 3.5.0-23-generic) using the Apache web server (v2.2.22), MySQL (v5.5.29) and PHP (v5.3.10) to manage the data as the back-end. HTML, CSS and JavaScript were applied to design web pages as the front-end language. The source code of the DRAMP website has been stored in the GitHub public repository. We are responsible for the continuous update, backup, error repair and interface optimization of DRAMP.

### Web architecture

#### Home page

The home page provides a brief preview and quick search of DRAMP. DRAMP features and relevant publications are presented. We record our regular updates on documents to show users what has been newly added and adjusted in the database.

#### Search page

There are two means provided on the search page for retrieval. Simple search is a quick and common way with which users can search the database in a specific field, such as peptide sequence, source, PMID and patent No. Advanced search is more precise, allowing for searches based on a combination of keywords from various fields, such as peptide sequence, length of sequence, source, 3D structure, activity, toxicity, modifications and so on.

#### Browse page

In the browser list, DRAMP provides many classifications according to different criteria for site visitors to select and browse the content that they are interested in.

The most important sorting is based on the data source type, resulting in non-overlapping partitions of the general dataset, patent dataset, clinical dataset and specific dataset. General data can be further viewed by taxonomy and activity, and specific data contain stapled and candidate AMPs.

#### Detail page

Every peptide entry in the database has a unique DRAMP ID and a detailed information page. The annotation scope provided varies depending on the dataset to which the entry belongs. For example, the general dataset displays general information, activity, structure, physicochemical information, comments and literature information. In DRAMP 3.0, stapled AMPs, a new part of the specific dataset, are presented similarly to the general dataset but more specifically to show their features, such as anchoring positions.

#### Tool page

DRAMP integrates ‘similarity search’ and ‘sequence alignment’ as tools to analyse users’ sequences in accordance with the database. The similarity search contains three sub-tools, namely, BLAST (33), Ssearch (34,35) and FASTA (36). BLAST can find regions of similarity between biological sequences. The program compares peptide sequences to DRAMP and calculates the statistical significance. Sequence alignment comprises global/local/multiple alignment using the analytic methods of Stretcher (37), Matcher (37) and Clustal Omega (38). All of these tools require sequence input in FASTA format.

#### Statistics page

Statistics of AMPs in DRAMP involving different aspects are displayed on this page, including the composition of the database according to the data type of AMPs, distribution of sequence length for general and patent peptides, amino acid compositions of peptides from the general dataset and top 8 AMPs for biological activity against common pathogens.

#### Download page

All the detailed data of peptide entries and the website original source code of DRAMP are freely available to users. In addition, URLs of some useful software are provided.

#### Quicklink page

Many links are shared to navigate to common resources, comprehensive databases, and bioactive peptide databases.

#### Help page

This page offers visitors detailed guidance to help better use the functions and tools integrated in DRAMP.

#### Submission page

This page is newly introduced in DRAMP 3.0. In order to accelerate gathering and integration of AMP information, DRAMP encourages researchers to share their known antimicrobial peptides based on published references or credible clinical trials. The website users can submit AMP data as much as they can, but at least including peptides sequence, literature title and PubMed ID/ Patent No./ClinicalTrials identifier. We check the content once a month regularly and complement the information of valuable sequences to meet the collection standard of DRAMP.

## RESULTS AND DISCUSSION

### New entries

**Figure 1.**
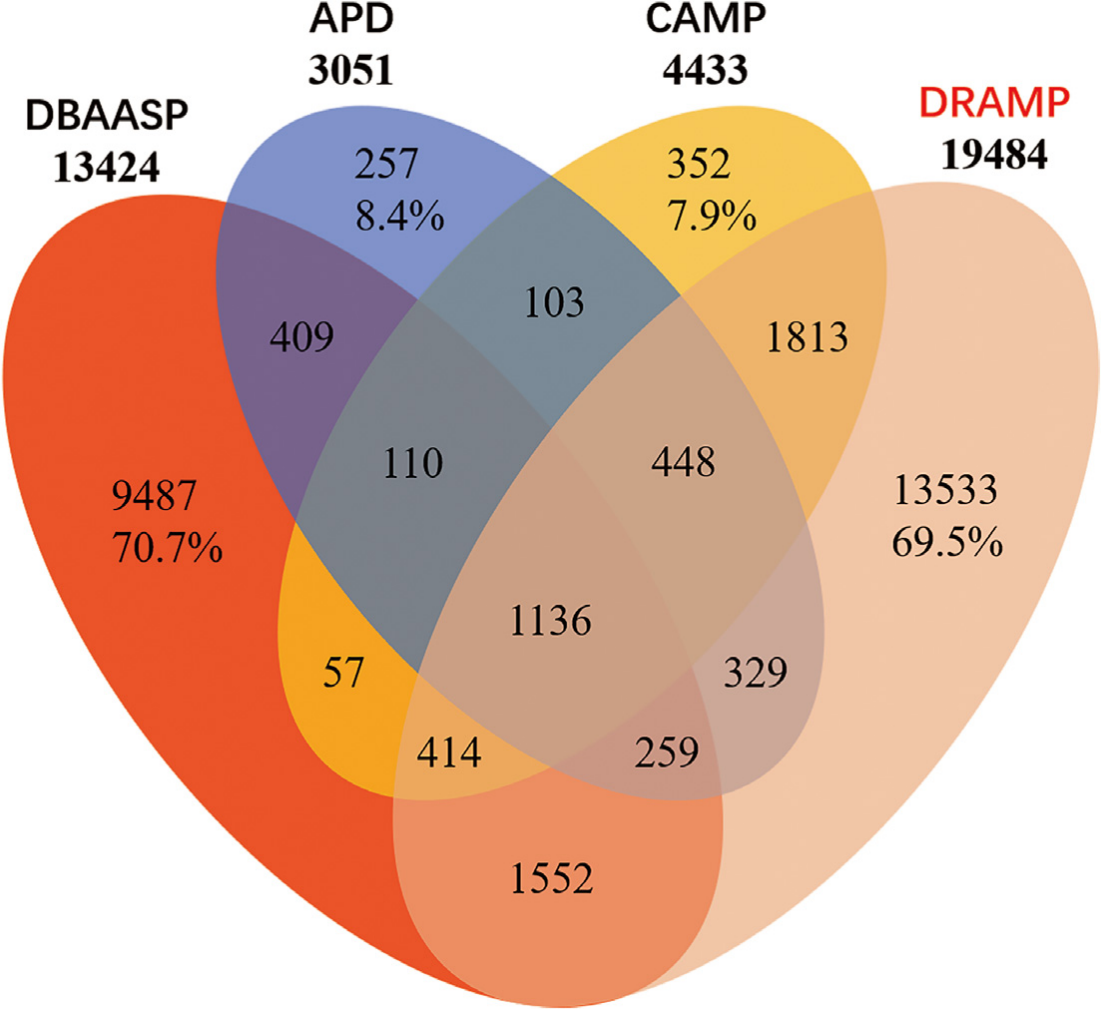
Venn diagram representing the numbers of overlapping and non-overlapping sequences from APD (https://wangapd3.com/main.php), CAMP (http://www.camp.bicnirrh.res.in/), DBAASP (https://dbaasp.org/home) and DRAMP (data as of 2021.1.1). The numbers of non-overlapping sequences in all four databases were calculated as percentages in corresponding areas. In the statistical process, we excluded sequences that were redundant, predicted or longer than 100 amino acids.

In this update, we entered non-repetitive AMPs from the literature and patents into our database, including some peptides with insignificant antimicrobial activity. DRAMP 3.0 has an increased 2360 major entries, including 805 general entries (101 natural entries and 704 synthetic entries), 1371 patent entries, 1 clinical entry, 181 stapled AMP entries and 5909 minor candidate entries since the last release. Most available databases, as well as DRAMP, have their own particular sequences and some overlapping sequences linked to other databases. We present the results of overlapping and unique sequences selected from APD, CAMP, DBAASP and DRAMP according to certain standards shown in Figure 1. DRAMP has 13533 nonoverlapping entries (approximately 69.5% of total), more than APD, CAMP or DBAASP.

### New classifications

#### Stapled antimicrobial peptides

**Figure 2.**
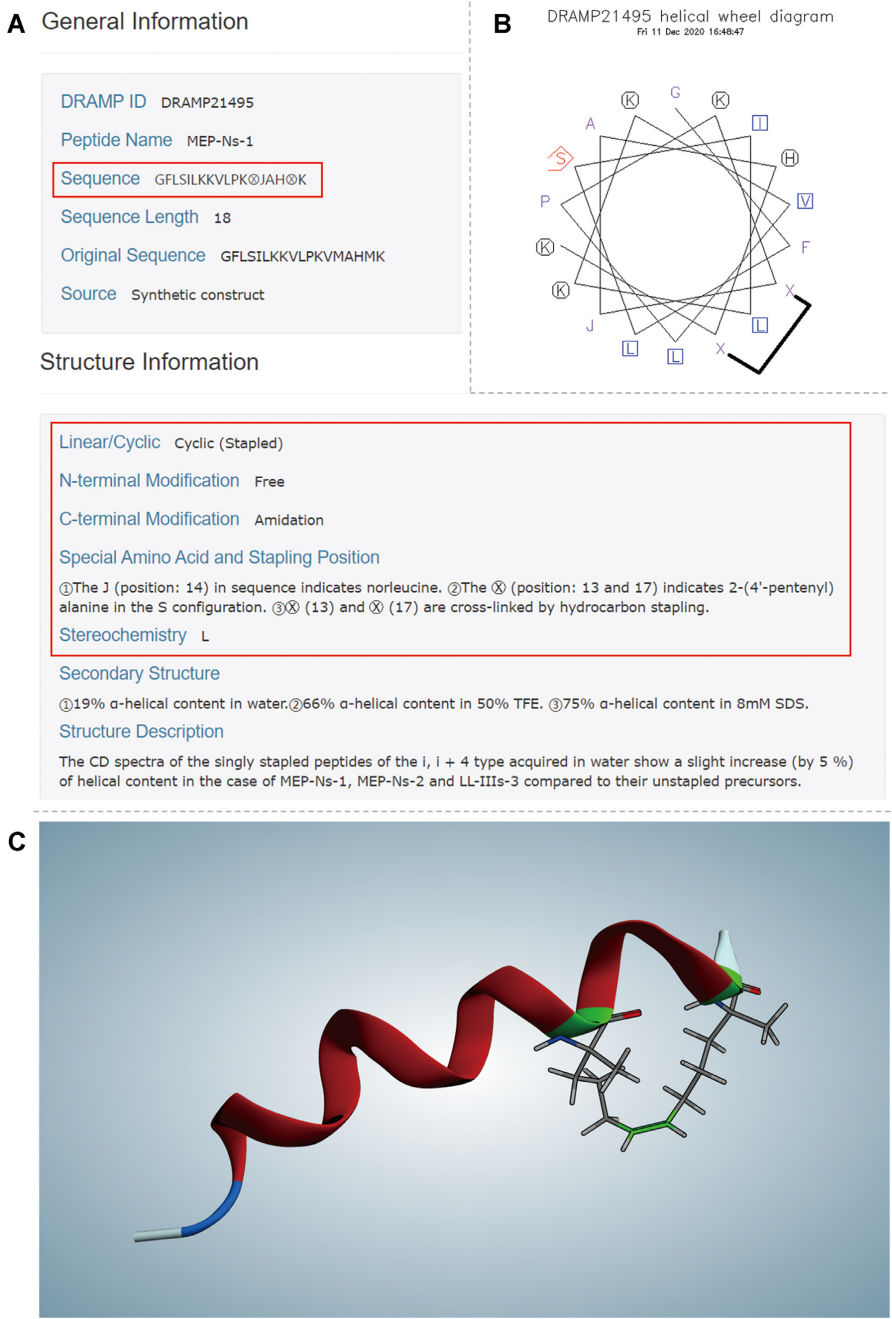
Screenshots taken from the page of entry DRAMP21495. (**A**) Enclosed alphanumeric 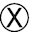
in the sequence is 2-(4′-pentenyl) alanine in the S configuration according to the description, which is the stapling amino acid. Such two amino acids are bridged by hydrocarbon stapling. In the red box is the discrepant part comparing with general entries. (**B**) Helical wheel diagrams of stapled peptides in DRAMP3.0. The ‘X’ represents stapling amino acid and the black fold line is the staple connecting them. (**C**) Visualization of 3D predicted structure of stapled peptides in DRAMP 3.0. The green ribbons represent the stapling amino acids, and the green bond is the bridge linking two residues of stapling amino acids.

Stapled AMPs prefer to enforce an α-helical structure due to artificial insertion of diverse staples, which helps to convey good antibacterial activity and strong proteolytic resistance (26). Stapled peptides are special and novel artificial modified peptides, so their information is presented in a more simplified and specific manner than general entries. Some inessential fields were removed, such as source, family, gene, UniProt entry and evidence code. Meanwhile, the original sequence and predicted structure diagram of stapled AMP are shown. We employ enclosed alphanumeric characters such as 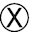 to represent stapling positions in sequence and included detailed comments on stapling amino acids, as well as stapling bonds (Figure 2A). In addition, due to the lack of an identified 3D structural diagram of stapled peptides, structural prediction may be an alternative way to realize structure visualization. In this paper, we used I-TASSER5.1 and MOE2019 (https://www.chemcomp.com/) to perform de novo or homology model of molecular structures, insert staples and minimize the conformational energy (39–41). In the third version of DRAMP, 181 helical wheel diagrams and predicted structures of all stapled peptides are present in the pages (Figure 2B and C), and relevant PDB format files can be downloaded without restrictions.

#### Candidate antimicrobial peptides

High-throughput screening and in silico methods establish an innovative approach to comparatively quickly design novel antimicrobials (42,43). To expand the retrieval scope and assist in the design of AMPs, we added thousands of candidate AMPs screened by a high-throughput platform. Such candidate AMPs are not included as real AMPs but just for reference at the moment because they have not been synthesized or assayed.

### New annotations

#### Cytotoxicity

**Figure 3.**
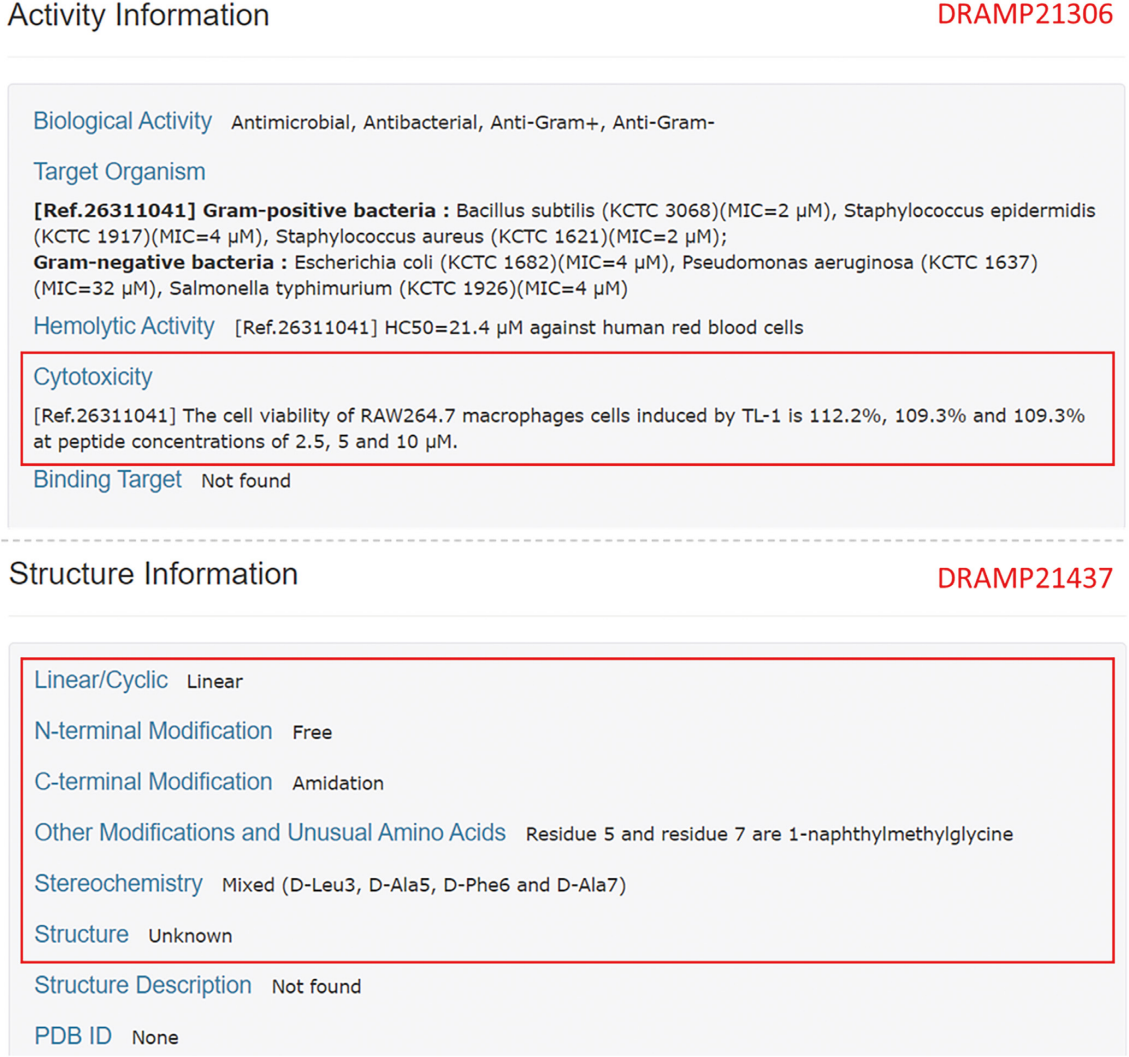
Visualization of the detailed activity and structure information page of DRAMP 3.0. The information specified in the red box is the updated fields and content. This screenshot was taken from DRAMP21306 and DRAMP21437.

AMPs have great antibacterial activity but are often associated with side effects in normal cells. These adverse effects limit the clinical application of AMPs. Customarily, toxicity against somatic cells, except red blood cells, is classified as cytotoxicity, which differs from haemolytic activity. Cytotoxicity is less investigated than haemolytic activity but also plays a supplementary role in reducing the side effects of AMPs. In this update, we added cytotoxicity information to DRAMP. Cytotoxicity information involves the PubMed ID of references cited, cytotoxic test results and corresponding somatic cells. The cytotoxic data mostly diagrammed in the original literature are embodied in the form of text by DRAMP, which supports statistical computing and analysis. An example taken from a page is shown in Figure 3.

#### Modifications and features

Many AMPs require modifications to function better in antibacterial effects, and some have their own features. The field ‘PTM’ was used to vaguely describe modifications occurring on peptides in the previous version, and it has now been extended to the five following fields: (i) Linear/Cyclic, (ii) N-terminal Modification, (iii) C-terminal Modification, (iv) Nonterminal Modifications & Unusual Amino Acids and (v) Stereochemistry. These make the description clearer and more intuitive. ‘Linear/Cyclic’ distinguishes between linear AMPs and cyclic AMPs according to whether there is a ring; ‘Nonterminal Modifications & Unusual Amino Acids’ lists what special or modified amino acids and intrachain bonds exist in the peptide chain; and ‘stereochemistry’ specifies whether the amino acids in the chain are L-type or D-type. The example is also shown in Figure 3.

#### Activity relationship map

**Figure 4.**
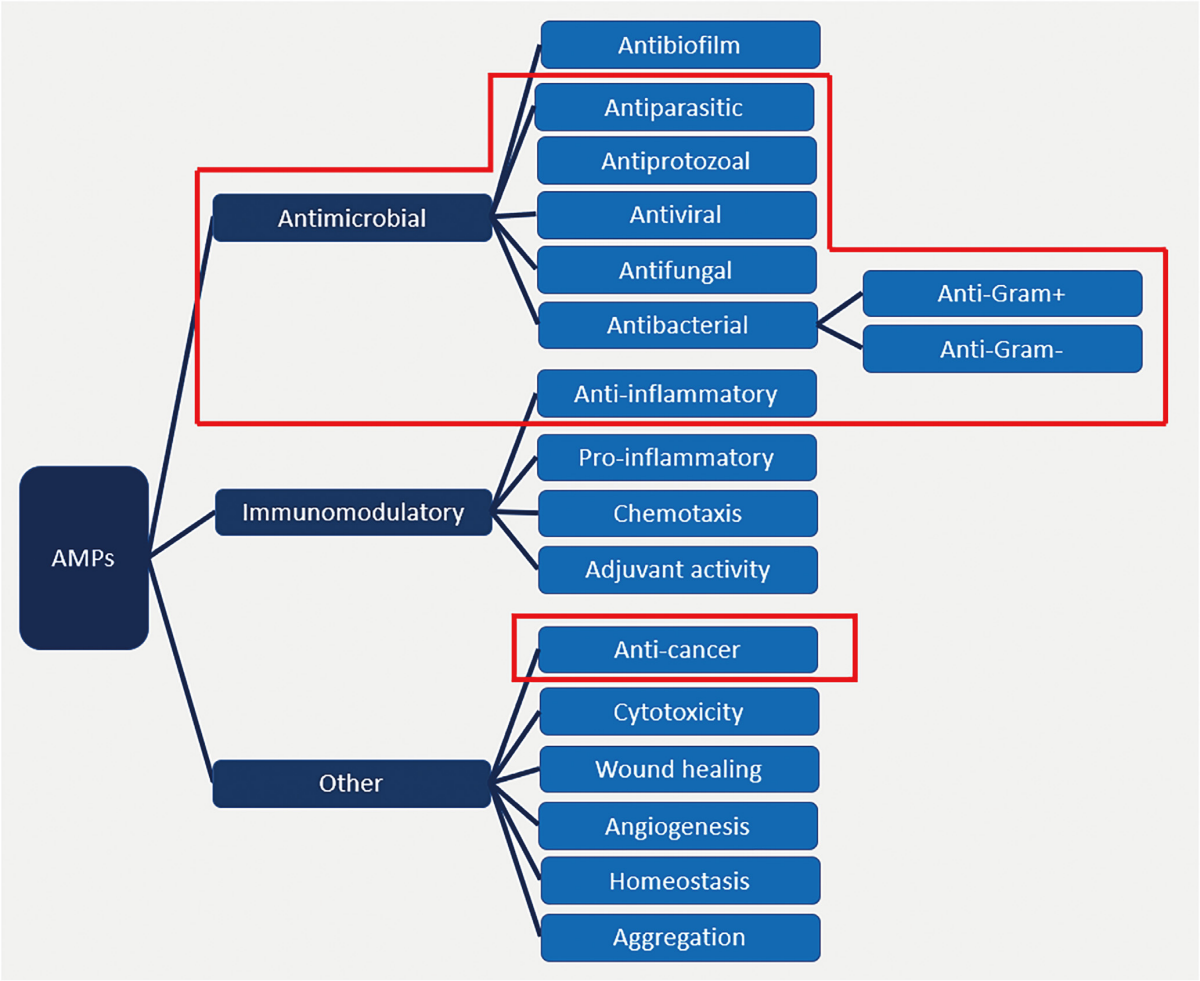
The relationship map of classifications by activity in DRAMP 3.0 summarizes known functions for AMPs. The section specified in the red box is the primary activities in DRAMP.

An increasing number of studies have investigated the multifaceted nature of AMPs. Recent work has shown that these molecules have strong potential not only as microbes but also as antibiofilm agents, wound-healing promoters, immune modulators, anticancer agents and anti-inflammatories (44–46). This prompted us to extend the activity scope of AMPs in DRAMP. The activity relationship map (Figure 4) summarizes almost all currently known biological functions of AMPs. Users can provide such activity keywords for retrieval or view the primary types of activity via the browse list page.

### Property of newly added stapled AMPs

**Figure 5.**
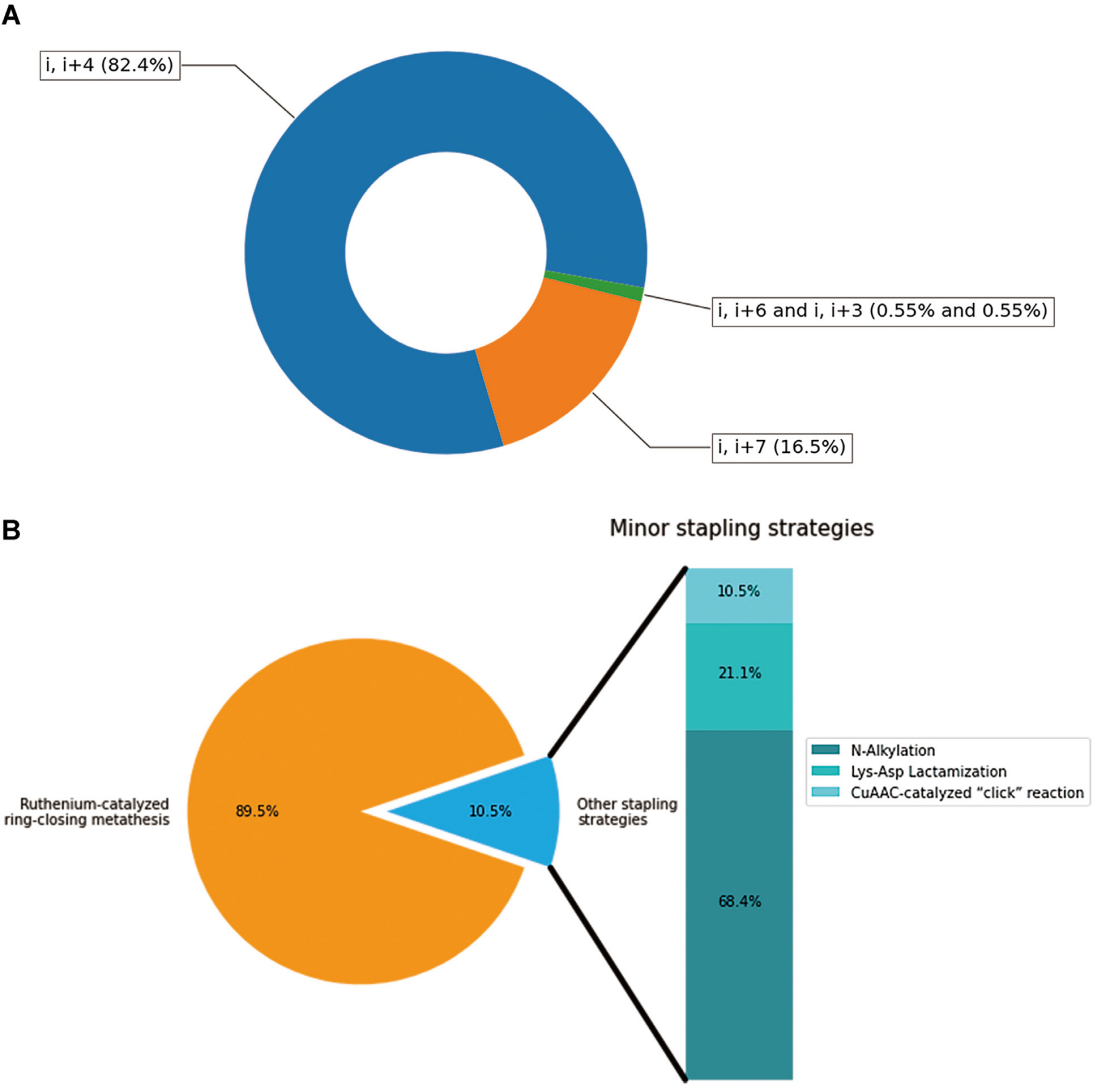
Analysis of stapled AMPs in DRAMP. (**A**) The pie chart presents proportions for different anchoring positions patterns of stapled AMPs. (**B**) The bar of pie chart presents proportions for stapling strategies used to synthesize stapled AMPs.

Analysis were conducted to show the sequence or structure property of the 181 newly added stapled AMPs. Generally, α-helix structure possesses 3.6 residues per turn on average, and the residues at positions *i*, *i* + 4, *i* + 7 and *i* + 11 are aligned on the same face (one, two and three turns, respectively); *i, i* + 3 is also available in some special cases for a single turn (47). The stapling positions analysis shows that (*i, i* + 4) and (*i, i* + 7) spaced residue pair are the most common anchoring positions patterns for stapling one or two helical turns among these positions, which account for 82.4% and 16.5%, respectively (Figure 5A). One of the most effective and popular stapling strategies for linear peptides is ‘all-hydrocarbon stapling’, which was first established by Verdine and co-workers using Ruthenium-catalyzed ring-closing metathesis (20,48). The method tethers the side chains of two nonnatural amino acids at the *i, i* + 4 or *i, i* + 7 positions, and the nonnatural amino acids are always *S-* or *R-*pentenylalanine (S5 or R5) and *S-* or *R-*octenylalanine (S8 or R8). The most applicable residue pair combinations in hydrocarbon stapling are S5-S5 and R8-S5. Stapled AMPs using ‘hydrocarbon stapling’ strategy account for the highest proportion (89.5%), while other minor stapling strategies like N-alkylation, Lys-Asp lactamization and CuAAC-catalyzed ‘click’ reaction take the last 10.5% part of staple AMPs syntheses (Figure 5B).

### Download links

DRAMP began with direct or potential help from other AMP databases. We are delighted to provide all DRAMP data, including all content in every field, without reservations for users to download and analyse. Therefore, we have completely uploaded the latest links to the DRAMP download page. The download page has been divided into four parts according to data type and different classification standards. The first part is classified by four datasets, including the general dataset, patent dataset, clinical dataset, and specific dataset. In the second part, our data are further categorized into 13 subgroups according to peptide activity or features. We also offer user-friendly free third-party software and tools involved in DRAMP, which can help researchers analyse our data and identify extending information. The last link is the external link to GitHub, where the source code of DRAMP is stored.

## CONCLUSION

The rational design of druggable AMPs targeting disease-causing organism shows great promise as well as obstacles, and large sets of well-annotated AMP data can contribute to the field. DRAMP is continually supported to provide reliable and detailed information about all kinds of AMPs with the aim of help researchers in this area. In DRAMP 3.0, there are 2360 major entries newly added compare with the previous version. Among these entries are 181 cyclic synthesized AMPs belonging to a novel particular type called stapled peptides, 3D structures of which were predicted using computational tools. We supplemented the cytotoxicity data of AMPs against normal human or animal cells on the basis of existing haemolysis data. Besides, five new fields are annotated, describing specific modification and sequence/structure features of entries. Updated statistics and shallow analysis have been released in the webpage in the form of charts and tables. More importantly, all data and annotations of DRAMP are accessible via download links. The update list of points in this version is shown in the Table 1.

**Table 1.** The update list of DRAMP 3.0 comparing to DRAMP 2.0

Update class	Classification	Subclassification		DRAMP 2.0	DRAMP 3.0
Entry	General dataset	Antibacterial	Anti-Gram+	1720	2452
			Anti-Gram-	1917	2618
		Antifungal		1553	1802
		Anticancer		90	149
		Antiparasitic		44	52
		Antiviral		212	215
		Antiprotozoal		17	17
		Antibiofilm		9	44
		Anti-inflammatory			4
		Pro-inflammatory			6
	Patent dataset			14739	16110
	Clinical dataset			76	77
	Specific dataset	Stapled			181
		Candidate			5909
Structure	PDB structure			272	283
	Predicted structure			82	263
Annotation field	Hemolytic activity			580	1470
	Cytotoxicity				63
	Linear/Cyclic				√
	N-terminal Modification				√
	C-terminal Modification				√
	Nonterminal Modifications & Unusual Amino Acids				√
	Stereochemistry				√
Webpage	Submit page				√
	Help & Statistics page				Updated

DRAMP will continuously collect new AMPs, re-update existing entries and add useful functions to facilitate rational design and research of AMPs.

## DATA AVAILABILITY

Users can access DRAMP 3.0 (http://dramp.cpu-bioinfor.org/downloads/) for the data. DRAMP is licensed under a Creative Commons Attribution 4.0 International License (CC BY 4.0) and allow download and use the data freely. An Excel document recording sequences from four databases, two Python scripts for statistics and drawing, and the original source codes of DRAMP are available in the GitHub repository (https://github.com/CPU-DRAMP/DRAMP-3.0).
